# Functional Assessment of Intermediate Vascular Disease

**DOI:** 10.1155/2018/7619092

**Published:** 2018-04-15

**Authors:** Teodora Yaneva-Sirakova, Ivanichka Serbezova, Dobrin Vassilev

**Affiliations:** ^1^Department of Internal Medicine, Cardiology Clinic, Medical University Sofia, Sofia, Bulgaria; ^2^“Angel Kanchev” University of Ruse, Ruse, Bulgaria

## Abstract

Interventional treatment in various vascular beds has advanced tremendously. However, there are several problems to be considered. We searched the literature and tried to analyze major parts of it. One is safety and applicability of coronary proven methods in other vascular beds. An unresolved problem is the functional assessment of intermediate lesions, as far as various target organs have quite different circulation from the coronary one and the functional tests should be modified in order to be applicable and meaningful. In the majority of the acute vascular syndromes, the culprit lesion is of intermediate size on visual assessment. On the other hand, a procedurally successfully managed high-degree stenosis is not always followed by clinical and prognostic benefit. In vascular beds, where collateral network naturally exists, the readings from the functional assessment are complicated and thus the decision for interventional treatment is even more difficult. Here come into help the functional assessment and imaging with IVUS, OCT, high-resolution MRI, and contrast enhanced CT or SPECT. The focus of the current review is on the functional assessment of intermediate stenosis in other vascular beds, unlike the coronary arteries.

## 1. Introduction

When we consider vascular beds, other than the coronary circulation, there are still debates over the main definitions, methods for investigation and clinical assessment, risk stratification, thresholds for intervention, and followup in intermediate vascular disease. Many answers are given in the current guidelines for peripheral artery disease, but there are still open issues to be addressed [1, 2]. When renal arteries are considered, the procedural success is more than 95%, but only 60–70% have clinical response, mainly blood pressure lowering or improvement in kidney function. Why do we have such a discrepancy and do we need to treat renal artery stenosis at an earlier time/stage? How exactly is clinical response defined for the cerebral and for the renal circulation? Or how do we define clinical response in the state of renal artery stenosis and stenting? How can we make functional assessment of other vascular beds? Is the traditional fractional flow reserve (FFR) applicable to them? Thus, naturally arises the question how is “hemodynamically significant” stenosis defined? There are differences in vascular reserve: for the coronary circulation, it is 4-5 times; for the renal circulation, it is above 20%; for the peripheral one, it may go up to around 80 times higher [3, 4]. Vasodilatory reserve depends on several factors such as endothelium-derived vasodilatory molecules, metabolites, sympathetic activity, and muscle mass (in the case of peripheral arteries) [3]. That poses questions about the applicability of coronary functional tests to renal and peripheral invasive functional testing. The substance receptors are region-specific and thus the response may be vasodilation in one microvascular bed and vasoconstriction in others. Do we need modification or region-specific tests? The discussion about the significance of intermediate vascular stenosis should include two aspects: (1) the clinical significance of an intermediate stenosis in the various vascular beds and its assessment and (2) the prognostic significance of an intermediate stenosis in the various vascular beds and its assessment.

*The aim* of the review is to summarize the current knowledge in the field of the functional assessment of intermediate vascular disease.

## 2. Materials and Methods

The literature was searched and articles from the beginning of the interventional treatment up to now were analyzed, to find that, despite the tremendous advance in interventional diagnosis and treatment, several problems, such as the clinical and prognostic significance of intermediate vascular lesions in other vascular beds remain to be addressed. More than 75% of the articles assessed were original articles, published in PubMed. Atherosclerotic renal artery stenosis and cerebrovascular and peripheral artery disease are the focus of the current review.

## 3. Functional Assessment of Intermediate Renal Stenosis

The treatment of atherosclerotic renal artery stenosis and fibromuscular dysplasia is quite different. The current review concentrates on atherosclerotic renal artery disease. It accounts for more than 90% of the renal stenosis cases. The relative frequency of the disease is around 50% in patient with known atherosclerotic disease [5].

In autopsy series studied by Holley et al. [6] the prevalence of renal artery stenosis (RAS) of more than 50% in patients above the age of 50 years was 27% and patients with diastolic blood pressure of more than 100 mmHg had 53% incidence. Between 2 and 5% of adult hypertensive patients have secondary causes [7]. Around 24% of the patients with resistant hypertension have significant RAS [8].

The most widely used indications for renal artery revascularization are [9] hemodynamically significant renal artery stenosis, accompanied by one or more of the following features:

- Uncontrolled hypertension
- Ischemic nephropathy
- Cardiac syndromes: flash pulmonary edema, uncontrolled heart failure, and uncontrolled angina

Despite the very good treatment options, interventional treatment of renal vascular lesions has not proved efficient enough [10]. One potential solution of the problem is interventional treatment based on functional assessment, because neither cineangiography in two projections nor duplex ultrasound has ensured the desired stenosis severity staging.

A major drawback in renovascular interventions is the absence of good discrimination of probable responders and nonresponders to stenting. The lack of clear definition may be due to difference in the thresholds for or the selection process for intervention for the different endpoints: renovascular hypertension or ischemic chronic kidney disease. Contemporary computer-tomographic or intravascular tomography has limitations in the assessment of the significance of RAS by using fixed planes and inability to assess the functional significance. An attempt to improve the selection process for responders was the use of functional assessment, either by interventional techniques or by incorporation of methods for cardiac computer-tomographic based FFR.

It should be noted that the renal and coronary circulations are quite different from one another. The heart is perfused in diastole and the perfusion pressure is largely dependent on the perfusion gradient between the epicardial arteries and the end-diastolic pressure. The kidney is perfused in systole at high pressure, that ensures high filtration rate. The high filtration fraction is a desired positive effect. However, this may lead to damage of the glomerular capillaries by the elevated pulse pressure [11, 12]. Another important aspect is that the renal perfusion pressure is dependent on the difference between the mean arterial pressure and the intra-abdominal pressure [13]. The commonly used vasodilatory substance for the coronary microcirculation – adenosine, has quite specific renal effect. Primarily vasoconstriction and after 1-2 minutes – vasorelaxation, due to the reaching a steady state plasma adenosine concentration and activation of A2AR receptors [14].

A landmark study was that of Mitchell et al. [15] which showed that FFR of 0.80 can be used as a threshold for functional significance of a RAS. The endpoint of the study was diastolic blood pressure lowering and it was conducted in patients with unilateral renovascular disease. Hyperemia was induced by papaverine. It is interesting to find if there is such a threshold for bilateral renal disease or when renal function, or systolic blood pressure, is the endpoint.

To define more precisely the group of responders to renal stenting, several biomarkers have been evaluated. Silva et al. [16] showed in 27 patients with refractory hypertension that initial brain natriuretic peptide > 80 pg/ml was a good predictor for positive clinical outcome after renal stenting. A meta-analysis of Ronden et al. [17] showed that neither serum creatinine decline after stenting nor pretreatment pulse pressure can be used as clinical correlation of successful intervention. The only positive result was for the diastolic blood pressure: the higher diastolic blood pressure pretreatment was, the lower it was after treatment. Similar correlation was found in patients with difficult-to-control hypertension, treated with renal denervation, mainly poorer response in patients with isolated systolic hypertension [18].

In ischemic nephropathy, a result from renoocclusive disease, a perfect angiographic procedure does not guarantee reversal of renal dysfunction. According to Rodriguez et al. [19], renal stenting in advanced chronic renal insufficiency has unfavorable clinical effect because of already developed nephroangiosclerosis. According to Vashist et al. [20], the diagnosis of renovascular hypertension can be made only when hypertension improves after stenting, because not all RAS are correlated with significant blood pressure elevation. This is also dependent on the stage of the RAS; in later stages exists no such correlation. Some authors postulate that continuous renin, angiotensin II, and aldosterone stimulation of the healthy kidney of a pair with unilateral stenosis may lead to perpetuation of the hypertensive and volume overload state even after revascularization [21]. Consequently, the question of the proper timing of the procedure is posed. The benefit of atherosclerotic RAS stenting is proved in patients with baseline normal or mildly impaired renal function [22]. Thus there may be a clinical benefit long before a renal artery stenosis becomes angiographically significant. That is why functional assessment of intermediate RAS rises as a question.

An attempt to assess the hemodynamic effect of RAS is made by several investigators with no uniform definition. It was first considered that a sufficient pressure gradient between the poststenotic part of the renal artery and the aorta is the leading factor for the significance of renal stenosis. There is consensus that a resting peak systolic pressure gradient > 20 mmHg is significant in RAS, but it was not clinically proven [23]. The utilization of a given pressure gradient has a significant drawback; mainly it is pressure dependent and thus in cases with very low systemic pressure it will be a too “low-flow, low-gradient” phenomenon. This can be overcome with renovascular dilatation, for example, under conditions of measurement of renal FFR. However, the outcomes from studies with renal FFR are not uniform. First, there is no consensus for the threshold (0.90 or 0.80); second for the agent which induces maximal hyperemia in the renal circulation (papaverine [24], dobutamine [25], and acetylcholine [26]), and third for the physiologic base of the utility of renal FFR. Renal FFR correlates well with other hemodynamic variables (resting mean pressure gradient *r* = −0.76; *p* = 0.0016; hyperemic mean translesional pressure gradient *r* = −0.94; *p* < 0.0001), but poorly with the angiographic severity of stenosis between 50 and 90% (*r* = −0.18; *p* = 0.54) [27]. In a study of 35 patients with difficult-to-control hypertension, stenting for RAS was done, with the following unexpected results [28]. Despite the strong correlation between Pd/Pa and transstenotic pressure gradient (*r* = −0.89, *p* < 0.001) on the one hand and renal FFR and transstenotic pressure gradient (*r* = −0.86, *p* < 0.0001) on the other hand, neither Pd/Pa nor renal FFR prior stenting was predictive for procedural success and blood pressure lowering [28] (*r* = −0.89, *p* < 0.001) (*r* = −0.86, *p* < 0.0001). Thus, there might be other factors that mark a potential positive effect after interventional treatment in RAS. In a study by Frauchiger et al. [29], the ratio between cortical end-diastolic velocity and peak systolic velocity was found to be an important predicting marker for procedural success after renal stenting.

Even mild renal artery stenosis can lead to increased renin release due to distal kidney hypoperfusion and thus elevated aldosterone and angiotensin II secretion [30]. They lead to morphological target organ damage. So, do we need earlier vascular treatment in order to prevent deterioration of target organ function? The clinical importance of renal stenting goes beyond blood pressure reduction, as far as angiotensin II and aldosterone lead to arterial stiffening, heart hypertrophy and fibrosis, brain vasoconstriction, and hypoperfusion [30]. Perhaps the target for evaluation should be renin concentration? Another consideration, when assessing the clinical significance of a RAS, should be the function of distal parenchymal vessels. Intravascular Doppler evaluation of the blood flow can aid in the assessment of the distal flow impairment relatively early [31] in comparison to other types of measurements, such as GFR or microalbuminuria. Kidney microvascular disease, often a result of arterial hypertension or abnormal renin production in response to uni- or contralateral atherosclerotic disease, may modify the effect of an intermediate RAS. The effect will be significantly more pronounced in the kidney with a stenotic artery [32]. The net clinical effects would be exacerbation of renal ischemia and upregulation of renin secretion in the presence of intermediate (angiographically) stenosis. Once the effect of the renal microcirculation is eliminated (almost eliminated), the large vessel stenosis can be adequately functionally assessed. There is also a debate on the method of renal pressure measurement.

There were trials, which try to find the best pharmacological agent to produce vasodilatation when measuring the translesional gradient of RAS. Protasiewicz et al. [33] compared the effect of dopamine and papaverine in a cohort of 14 patients with moderate RAS. The result was that the postlesional systolic pressure (Pd) after papaverine was not changed significantly in comparison with dopamine. Thus, the intrarenal dopamine administration resulted in significantly higher translesional gradients than papaverine infusion. A trial with similar endpoints and aim was conducted with acetylcholine as a hyperemic agent [26]. Acetylcholine produced effective vasodilatation and elevation of translesional pressure gradient in intermediate stenosis. 20 mmHg was used as a threshold for intervention. The result was a significant drop of systemic blood pressure at least 30 days after dilatation.

The difference obtained with the use of different hyperemic agents is due to their differential effect on their specific culprit vessels: the microvasculature for papaverine and the renal artery for dopamine. That translates into different effects on the postdilatation measurement of the translesional renal gradient (Pd/Pa) which should be born in mind when performing an FFR based renal intervention.

Renovascular hypertension and renal dysfunction are due to obstruction of the blood flow in resting conditions, unlike myocardial ischemia, which is provoked under hemodynamic stress. That has led some authors to the idea that resting ratio of distal/proximal renal pressure is enough to assess the effect of a lesion [34]. De Bruyne et al. [35] showed that distal to proximal ratio 0.90 was the threshold that was clinically meaningful for a significant rise in renin secretion. As this has not been proven in other studies, a certain threshold of distal pressure to aortic pressure is still investigated. Drieghe et al. [10] proved that there was a fairly good correlation between angiographic, Doppler, and pressure (Pd/Pa) measurements of renal arteries, but still 38% of the stenoses > 50% were falsely significant with a velocity > 180 cm/s in 55% of the cases.

There are some more differences between the coronary and the renal circulation. RAS produces constriction of the efferent arteriole in an attempt to preserve the pressure gradient through the glomerulus and as a consequence flow is reduced. In the heart, an epicardial stenosis produces vasodilatation in an attempt to keep the flow, but this reduces pressure distally to the stenosis. This is why there is no consensus statement for the threshold of clinical significance of renal FFR; for example, Kapoor et al. proposed 0.90 [36] while Mitchell et al. proposed 0.80 [15].

Potential drawback in the proper measurement of renal FFR may be significantly elevated central venous pressure such as in decompensated heart failure. The proper estimation of renal FFR is guaranteed in cases of very low central venous pressure [27].

To conclude, when discussing the significance of RAS interventions, it is important to clarify the clinical endpoints in the light of medical treatment as well. There are several clinical endpoints that can be used in the assessment of the effect of interventional treatment of RAS. Blood pressure reduction, measured in mmHg, is only a small part of the potential benefits. Other beneficial effects, which are not discussed, are the reduction of the number of medications that are used for treatment and the slowing of the renal function deterioration [37]. The renal FFR is important in the assessment of the functional significance of renal artery stenosis. Lack of significant transstenotic pressure gradient in morphologically significant stenosis can be encountered in the case of slow flow due to renal parenchymal disease [38].

Only the combination of imaging, functional, and laboratory biomarkers can effectively define probable responders to renal stenting.

## 4. Functional Assessment of Intermediate Cerebral Stenosis

Major problems to be considered in the assessment of cerebrovascular disease and before carotid stenting are the functional significance of intermediate stenosis or of tandem lesions of a single artery [39, 40]. Carotid plaque morphology, composition, and severity are correlated with cardiovascular mortality [41, 42]. In a study by Petersen et al. [43], the mortality in patients with intermediate and stenoses above 75% was one and the same, lower than nearly occlusions or total occlusions, but higher than that in patients without or with minimal stenosis. Some authors [44] postulated that a potential destabilizing factor in intermediate carotid stenosis might be platelet function. What is the clinical significance of FFR in intra- and extracranial lesions as far as cerebral microcirculation works at its nearly maximal vasodilatory state? In the case of bilateral carotid stenosis, even of intermediate size, there is a high risk for steal phenomenon, which should be assessed dynamically and not only in resting state. It is accepted that an elevated need for blood supply in the state of carotid stenosis may cause brain hypoperfusion; thus, even nonsignificant stenosis should be assessed.

It is widely known that 40% of recurrent strokes occur in patients with intermediate 50–69% stenoses [45, 46]. Thus, their hemodynamics and its modification by collaterals, other significant stenoses, and the characteristics of the plaquesare of high importance when considering an intra- or extracranial stenosis for intervention.

As there is a CT FFR assessment of coronary arteries, respectively, a CT FFR of the carotid arteries is underway [47].

### 4.1. The Role of Collaterals for the Significance of a Stenosis in the Brain Circulation

The circulation of the brain consists of several circuits. The main one is the circle of Willis. The inflow arteries of the circle of Willis are the internal carotid arteries, the basilar artery, which is formed by the left and right vertebral arteries, and the ophthalmic artery, which is a branch of the internal carotid artery and is connected via collaterals to the external carotid artery and thus the common carotid artery. The outflow arteries are the anterior, the middle, and the posterior cerebral arteries [48]. Sufficient stenosis in one of the inflow arteries is overcome functionally by compensatory enhanced flow in the other arteries. This might not be the case in the simultaneous presence of a severe stenosis in other arteries of the circle. The presence of good collateral flow has several clinical implications [48]. First of all, it modifies the pressure drop of a stenosis. If the stenosis of one of the arteries that flow into the circle of Willis is not balanced with collateral flow, this will lead to very high intrastenotic velocity and extreme poststenotic drop of pressure [49]. The elevation in the flow velocity in the stenosis is a compensatory mechanism to achieve proper supply to the distal part of the circulation. On the other hand, it stabilizes the most stenotic part of the plaque by promotion of fibrous cap growth. The upstream slope is the most prone to intraplaque hemorrhage [50], because of the abrupt and significant pressure drop [51] downstream which leads to elevation of the shear stress in the plaque and elevated tensile stress in the proximal part and a consequent unloading of this pressure with rupture of the plaque [52–54]. Cicha et al. [55] found that in the majority of the cases (86%) rupture took place at the upstream side of the plaque. They observed endothelial erosions more frequently downstream the blood flow. Thus, two stenoses with one and the same percentage will have different functional significance on the basis of presentation or absence of collateral circulation. The one with collateral flow will be relatively more stable, with lower pressure drop and lower intraplaque flow velocity in comparison with the unprotected one. The percentage of luminal stenosis should not be the sole standard for revascularization. These findings, however, are based primarily on theoretical calculations. An attempt to find their clinical implications was made in several studies. Meairs and Hennerici [56] studied the atheroma of 22 symptomatic and 23 asymptomatic patients with 50–90% internal carotid artery stenosis. Plaque surface motion during one cardiac cycle was recorded with 3D ultrasound. Symptomatic plaques demonstrated inherent plaque movement.

If we consider patients in the acute phase of stroke and aim at revascularization in this precise group, the role of collaterals could be even more significant, as far as the absence of, for example, pial collateral branches to the region at risk significantly increases the hemorrhage risk.

### 4.2. The Effect of a Significant Plaque on the Flow on the Contralateral Plaque

In case of stenosis in one of the cerebral arteries that are components of the Willis circle, the flow through the corresponding contralateral circulation will compensate the flow in such a way so as to maintain proper flow rate (around 4.15 ml/s) to the brain area at risk. If the contralateral side is affected with plaques too, the flow could not be increased [48]. Compensation and maintenance of cerebral blood flow can be achieved with an increase in diastolic flow or with prolongation of the systolic time. The maximal flow in stenotic, for example, carotid arteries, occurs much later in the systolic time curve in cases of absent contralateral flow more than if the contralateral flow is preserved. Pulse delay in a stenotic extra- or intracranial artery is a reliable marker of contralateral stenosis [48].

### 4.3. The Role of Plaque Morphology for the Significance of a Stenosis in the Brain [57, 58]

The principles that affect blood flow through and out of a stenosed artery are mainly two: Hagen–Poiseuille (pressure gradient in a tube with laminal Newtonian flow) and Bernoulli (maximal pressure drop if there is sudden expansion at the exit of the stenosis, so that part of the kinetic energy is dissipated within the turbulent flow). If the morphology of the stenotic plaque is one with gradual downslope at the distal end, the basic principle that is applied when assessing the pressure drop is Hagen–Poiseuille; the pressure drop is proportional to the length of the stenosis. If the stenosis is with an abrupt ending, a pressure drop that is due to turbulent flow and that was assessed by Bernoulli equation will be added to the pressure expected only in the Poiseuille equation [59]. This has two clinical implications: first, the morphology of the plaque is of critical importance for the stability as far as its stability is lower in the presence of larger pressure drop such as in abrupt ending stenosis and consequently turbulent flow; second, turbulent flow leading to larger pressure drops produces audible murmurs. Thus, one and the same percentage of stenosis with audible murmur, that is, a clinical equivalent to turbulent flow and consequently to ultrasonographically visualized abrupt end, will be much more unstable and of much higher functional significance because of the larger pressure drop that destabilizes it.

According to the Laplace's law a greater tension is applied to the caps of milder plaques than to those of more significant stenoses, under one and the same intraarterial blood pressure and cap thickness [60]. This can be the potential explanation for the higher risk of intermediate plaque disruption than that of more severe stenosis [60]. As in the coronaries, the morphology of the intracranial plaques is of significant importance for their stability and risk of rupture [61]. MRI has emerged as the gold standard for the morphologic assessment of cerebral plaques [62].

Resting cerebral blood flow has no correlation with the degree of the internal carotid artery stenosis [63]. The gold standard for functional assessment of cerebral blood flow reserve is the acetazolamide challenge on SPECT imaging. There are three types of acetazolamide responses during hemodynamic challenge: (1) normal initial flow and flow augmentation after acetazolamide; (2) low flow before and flow augmentation after infusion; (3) low or normal flow initially and no augmentation or decreased flow after acetazolamide (Diamox) [64]. The first response is the normal response in a normal cerebral circulation. The second is the normal response of cerebral blood flow in a patient with cerebral stenosis and preserved vasodilatory reserve. The supply is enough in the resting state but under increased needs it is not enough. The third case is encountered in patients with cerebral vascular stenosis and strained-to-the-limit vasodilatory reserve. They are at an increased risk of serious stroke and need emergent intervention [65]. This test may be of high importance for the proper risk assessment in patients with diffuse atherosclerosis, to whom staged procedures are needed. On the other hand, strained-to-the-limit vasodilatory reserve may lead to impaired autoregulation. This can imply a significant hemorrhagic postprocedure (stenting of endarterectomy) risk for the patient [66].

When discussing cerebrovascular lesions, the fact that there is no correlation between the degree of the stenosis and stroke risk should be emphasized [66]. Visually or functionally intermediate carotid stenosis may be vulnerable. Other factors, such as irregular plaque surface, thickness, and ulceration on ultrasonographic surveillance [67], may play a more important role. However, a more precise estimation of plaque vulnerability in intermediate vascular lesions can be given with IVUS, OCT, MRI, SPECT, and PET. Gupta et al. [68] found that intraplaque hemorrhage, lipid-rich necrotic core, and thinning of the fibrous cap on MRI were highly predictive of future stroke.

Options for other types of functional imaging are underway. They include inflammation, proteolysis, and thrombosis. Inflammation is a key destabilizing factor in the pathogenesis of atherosclerotic plaques. It is considered that molecular imaging of intraplaque inflammation severity may predict plaque vulnerability [69]. FDG-PET is the most conventionally used tracer that is readily taken by the macrophages in unstable plaques [70]. Another pathway involved in plaque destabilization is proteolysis. Matrix metalloproteinases and cathepsin cysteine proteases destroy the fibrous cap and destabilize the plaques. Their concentration is proportional to the proteolytic process and can be assessed quantitatively by radiolabelled inhibitors with successive SPECT or PET imaging [71, 72]. Elevated thrombomodulatory factors concentration and activity play a key destabilizing role [73] and are proven significant in 74% of the patients with stroke and in 35% of those with transitory ischemic attacks. Thrombus formation can be visualized with high-resolution MRI [74].

It can be summarized that contemporary imaging options with SPECT, MRI, and PET can provide functional assessment in the face of intermediate plaque instability. Current guidelines [75], however, exclude interventions in nonsignificant or intermediate asymptomatic stenosis.

## 5. Functional Assessment of Intermediate Peripheral Vascular Disease

One major difference between the coronary and the peripheral circulation is that in the peripheral arteries pressure drops only when the luminal flow is reduced with more that 75%. At this stage, the resting flow is already impaired and the treatment is difficult [76].

Endovascular treatment is a first-line therapy in the contemporary treatment of peripheral vascular disease [77]. In the everyday practice postexercise ankle-brachial index (ABI) can be a good marker for inducible claudication. However, a limitation in its use is the lack of universally accepted loading protocol. This makes the results hardly comparable. ABI is also not applicable in cases with noncompressible vessels and in some cases with proximal peripheral artery disease with good distal collateralization. Options to solve the problems are exercise oximetry and exercise near-infrared spectroscopy [78, 79]. The clinical significance of an intermediate peripheral vascular stenosis depends on its localization, collateralization, and the flow reserve of the underlying skeletal muscles at maximal vasodilatation during exercise or with adenosine infusion [76]. A study with contrast enhanced ultrasound of limb skeletal muscle in dogs was conducted to show the physiologic effects of large and small vessel diseases and collaterals on the muscle perfusion during exercise or adenosine infusion [76]. Similar studies were conducted in patients with claudication Rutherford classification grade I, category III [80], and classes I–III [81] with the result that peak perfusion and time to peak perfusion in claudicants were similar to those in the controls, but with no increase after exercise. Potential advantage of the method is the simultaneous functional assessment of the macro- and microvasculature of a certain muscle group. Another potential solution of problem is the use of peripheral FFR in intermediate stenosis. Hioki et al. [82] found a good correlation of significant strength (*r* = 0.857, *p* < 0.001) between intravascular peripheral FFR with papaverine and postexercise ABI. The correlation between the peak-to-peak pressure gradient in hyperemia and postexercise ABI was also significant, but of relatively milder strength (*r* = −0.626, *p* = 0.013). Thus peripheral FFR seems to be more precise method for the assessment of intermediate lesions than peak-to-peak gradient [83]. It can be applied under all conditions and without the restrictions of a standard treadmill protocol. Some authors produce hyperemia with intravascular isosorbide dinitrate [83]. They measured mean pressure gradient and mean pressure ratio at baseline and after 250 mcg intraarterial isosorbide dinitrate in 23 lesions of intermediate size in the iliofemoral segment. Hyperemic mean pressure gradient was considered respective of the peripheral FFR. The test was controlled with ultrasonographic measurements of peak systolic velocity ratio (velocity at the point of maximal stenosis divided by the velocity in the closest adjacent healthy vessel) 30 days before the intravascular measurement. A threshold > 2.5 was considered significant for the measurement of peak systolic velocity ratio on Doppler ultrasound. Hyperemic mean pressure gradient and hyperemic mean pressure ratio were proved to be significantly correlated with peak systolic velocity ratio with an optimal cutoff value for peripheral FFR 0.85, equivalent to peak systolic pressure gradient > 2.5. However, the problem with peripheral FFR procedure and readings in complex lesions (as is the case in most patients with peripheral artery disease) remains open and needs to be studied in depth. The prognostic significance of FFR guided interventional treatment of intermediate stenosis in the peripheral arteries also needs further assessment.

## 6. Functional Assessment of Intermediate Mesenteric Stenosis

There are just a few case reports for the probable significance of FFR in mesenteric disease [84]. The splanchnic circulation and its response to vasodilating stimuli is poorly understood and understudied. From theoretical point of view maximal hyperemic state reproduces postprandial hyperemia and may be indicative of ischemia in the state of angiographically intermediate stenosis [85].

## 7. Conclusion

Functional assessment guided interventions are the current gold standard for coronary revascularization. The basic principles of them can be applied to other vascular beds, with the note that there are organ specific responses that should be accounted for. Further functional studies are needed with combination of imaging and specific biochemical markers of target organ ischemia due to intermediate vascular lesion. The intermediate stenosis under loading conditions may be exposed to quite different factors and may be easily destabilized. The clinical significance of such functional tests for assessment of intermediate vascular stenosis is self-evident. But standardization and proof of prognostic significance are still lacking.

## Abbreviations

ABI: Ankle-brachial index
CFR: Coronary flow reserve
CT: Computer-tomographic
FFR: Fractional flow reserve
GFR: Glomerular filtration rate
IVUS: Intravascular ultrasound
MRI: Magnetic resonance imaging
OCT: Optical coherence tomography
PET: Positron-emission tomography
RAS: Renal artery stenosis
SPECT: Single-photon emission computed tomography.
